# Introduction to using symbolic regression for interpretable machine learning in healthcare

**DOI:** 10.1038/s43856-026-01914-x

**Published:** 2026-10-09

**Authors:** Michael Ferguson, Feifan Liu, Honghuang Lin, David D. McManus, Ben S. Gerber

**Affiliations:** 1 Department of Medicine, University of Massachusetts Chan Medical School, Worcester, MA USA; 2 Department of Population and Quantitative Health Sciences, University of Massachusetts Chan Medical School, Worcester, MA USA

**Keywords:** Prognosis, Diagnosis

## Abstract

The past decade has seen machine learning play an increasingly important role in medicine. However, many of the state-of-the-art models being developed and deployed suffer from a lack of transparency, limiting integration into the clinical decision-making process and raising concerns about patient safety. Here we call attention to symbolic regression, a largely overlooked machine learning technique that aims to be both accurate and interpretable by learning readable mathematical models from data. In recent years, it has undergone rapid advancements with successful application to problems across a wide range of disciplines. In this perspective, we provide an approachable introduction to symbolic regression, highlight its strengths and limitations, and explore its potential applications in medicine.

## Abstract (web summary)

Ferguson et al. provide an approachable introduction to symbolic regression, a machine learning technique which learns human readable mathematical models. They also comment on its potential applications to medicine.

## Introduction

Symbolic regression (SR) has received increasing attention in recent years^1,2^. This interest may be derived from recognizing that machine learning (ML) models not only need to be accurate and demonstrate high performance, but also be interpretable^1^. SR provides prediction models that are compact and “white box” in nature (transparent with the ability to inspect and understand how the model generates its predictions). While there is a common perception that a necessary tradeoff exists between accuracy and interpretability, this may not apply to SR. In optimal circumstances, SR can produce models with both interpretability comparable to classical regression models and performance competitive with state-of-the-art tree-based and deep learning models^1,3^. In healthcare, ML implementation incorporating SR may offer opportunities to enhance performance while maintaining transparency and patient safety^4^. However, its value over other ML approaches remains unproven.

Outside of medicine, SR has been widely applied in various scientific and engineering disciplines, with physics being the most common^1,2^. When physical laws and theorems can be formulated mathematically, it enables physicists to apply mathematical reasoning to solve or simulate problems far beyond the original scope of the stated results (e.g., Newton’s law of gravity)^5^. For this reason, a black-box model of a problem has far less utility than a mathematical one. This makes SR a natural choice for physicists hoping to learn natural laws or mathematical models from data^6^. Additionally, empirically learned laws are natural choices for testing SR algorithms. A database of equations used during Richard Feynman’s lectures is commonly used as a benchmark for SR algorithms’ capabilities for exact equation discovery^1,7,8^.

SR has been explored in other mathematically oriented fields. In economics, SR produced an analytic metric for credit scoring, providing insights in credit risk evaluation mechanisms^9^. In climate research, SR created a novel surface resistance parameterization^10^ and has been applied to wind speed forecasting^11^.

Interestingly, these other domains use medical datasets as benchmarks for newly developed SR algorithms. Approximately one-third of datasets used in SRBench (a framework for transparently testing performance of SR models) are related to medicine, including heart disease, breast cancer, and thyroid disease. Additionally, in the SRBench 2022 competition, explainable models for COVID-19 cases, hospitalizations, and deaths were explored on real-world data. However, awareness of SR as an available technique to apply to healthcare data remains low. To our knowledge, there is a lack of introductory publications discussing its readiness for use as a practical tool by the medical informatics community. In this perspective, we describe what SR is, its benefits and limitations, and how it can offer value to healthcare data modeling.

## What is symbolic regression (SR)?

Formally, SR is a machine learning framework designed to discover symbolic mathematical expressions from data that optimizes a given objective function^8,12,13^. In a simplistic example, an SR model trained on data obtained from measuring mass (*m*), acceleration (*a*), and force (*F*) of a moving object can identify “*F* = *m* × *a*” as the optimal function. This approach balances symbolic complexity (e.g., number of terms or operators in an equation) and a loss function (i.e., describe how well the formula fits the data) to ensure both interpretability and predictive accuracy. Unlike traditional regression methods that assume a predefined functional form, SR explores a vast space of possible expressions built from simple user-defined primitive functions (e.g., addition and multiplication, exponentials and logarithms, and trigonometric functions). The goal of SR is to identify (or approximate) an idealized model consistent with the underlying mechanisms of the system being modeled. It accomplishes this by constructing the model’s functional form dynamically and optimizing a small number of constants, rather than relying on many weights and nested simple functions, as is the case with black-box networks.

SR is frequently dominated by a genetic programming (GP) approach. These algorithms replicate the process of natural selection, with competition among candidate formulas (“survival of the best function”). While there are many highly sophisticated genetic algorithms for training SR models, a foundational approach^1,13,14^ can be described as follows. Initially, a population of formulas is created at random. Evolution occurs by iteratively repeating four major steps: selection, crossover, mutation, and optimization of constants. Selection involves choosing which equations in the population will survive to the next iteration, with the fitness of an expression (typically negative loss) determining its probability of surviving. During crossover, for each expression that did not survive, pairs of the remaining functions are selected to create child expressions by swapping pieces of their expressions. Next, a mutation occurs, with each child having pieces of its expression replaced at random. Finally, there is a probability that constants are optimized. This process is then repeated iteratively until the user-defined stopping criterion is satisfied (e.g., maximum iterations or target loss).

The second most popular style of SR algorithm utilizes neural networks (NN) for training, where several approaches exist. The least complicated approach is the equation learner, which represents the function as a NN whose activation functions at each layer consist of all possible primitive functions. Training involves adjusting the weights using gradients (standard training for a NN) to construct a symbolic representation. This technique is preferred when high performance and scalability are needed more than interpretability. A second approach is to learn functions through deep reinforcement learning (DRL). This process represents expression trees as a sequence of tokens and trains a recurrent neural network (RNN) agent via reinforcement learning to learn a policy for constructing the expression sequentially. This technique is preferred when there is a need to constrain the search space of equations, incorporate domain knowledge more effectively, or directly intervene in the training process^1,15,16^. Finally, a third approach is to use transformers to output tokens representing equations from datasets. These transformers are pre-trained on datasets generated from a wide variety of mathematical equations using token-level cross-entropy (with the true function as a loss function). In doing so, the equations are learned through network inference, eliminating the need to construct equations via a long training process for each dataset. This makes it desirable when speed and scalability are needed, but less so when desiring flexibility or using data that is difficult to represent using the pre-training equations^16,17^.

Finally, there are alternative SR models created to address some limitations faced by standard SR techniques and their variants. For example, Alaa and van der Schaar^18^ represent the target function as a NN similar to an equation learner, but introduce a variation. Instead of relying on the inclusion of primitive functions in each layer, they instead use Meijer-G functions for activation functions. Meijer-G functions form a general class of functions that cover polynomials, exponentials, logarithms, trigonometric, and other functions. Which function they represent is entirely determined by a set of numerical hyperparameters. By computing gradients of the loss function with respect to these parameters, the model can learn primitive functions directly rather than relying on user-defined specifications. This allows for the functional form of the full equation to be determined via numerical optimization instead of the usual trial-and-error approach.

Jin et al. describe a Bayesian statistical approach to SR^19^. Functions are defined as trees where the response variable is a linear combination of trees. Informative priors to function parameters (e.g., constants) are provided. The SR model then represents the posterior distribution of the underlying tree structure, which is computed using Markov Chain Monte Carlo methods. This provides statistical uncertainty estimates for the learned function, incorporates prior knowledge, efficiently explores the large space of possible equations, and can produce simpler expressions than GP.

## What are the benefits of SR?

### Practical application

Several software packages are available for SR implementation, which provide a streamlined user focused experience. For genetic algorithms, PySR^13^ offers a highly flexible and customizable open-source Python tool with a robust API that provides tuning and workflow tips for users (the backend uses a highly optimized Julia library). For neural network algorithms, Deep Symbolic Optimization (DSO) offers an actively developed unified framework for deep symbolic learning (including SR)^20^ and provides an sklearn-style interface for ease of use. SR models utilizing such packages may be trained with only a few lines of code, with the ability to review candidate equations during fitting as well as select a specific expression for prediction.

### Integration of domain knowledge

Despite its mathematical nature, SR does not require a priori knowledge about the data (though Bayesian approaches can be taken)^19^. While domain expertise and mathematical knowledge offer advantages, SR is an entirely data-driven process like other ML models. Hence, it requires no specialized knowledge to use.

Alternatively, SR can use domain knowledge. Usually, derived model functions only include pre-defined specific primitive functions. Therefore, by utilizing domain knowledge, one can build models including only the most relevant and meaningful primitives. For example, when studying growth dynamics, generalized logistic models known as hyperbolastic models are often used^21^. By including inverse hyperbolic sine, the exponential function, square, square root, and hyperbolic tangents into the set of primitive functions, the search space (set of possible equations discoverable during training) will include relevant hyperbolastic models and similar variants.

### Interpretability

Given that an SR model takes the form of a mathematical formula, results can often be interpreted by their symbolic representation. It offers the ability to manually compute and understand output, predict behavior from distribution data, and glean insight into the underlying mechanisms of the process being modeled. This offers a great advantage over solely relying on the explainability tools and interpretability metrics used with black-box models (e.g., SHAP), which have little utility outside of qualitative analysis and may not be consistent or reliable^22,23^. Also, it offers insights into generalizability. For example, consider the case when SR performs well, but its formula indicates a relationship that runs contrary to established knowledge. This may suggest that there are issues or anomalies with the training data (noise, irrelevant features, sampling differences, or non-representative data), which would cause the model to generalize poorly on new data.

It has been noted in the data science literature that for any given data set, many different models will provide near-optimal performance (the Rashomon effect^24^). Liu and Rudin define a data set’s Rashomon set as the set of all mathematical functions (i.e., trained models) achieving a goodness of fit within acceptable bounds^24,25^. The task of constructing interpretable models can then be reframed as discovering the most desirable and interpretable functions within the Rashomon set. They argue that this reframing allows us to be bold in our pursuit of interpretability and that we should not shy away from imposing model constraints, such as sparsity. Furthermore, they envision this search as an interactive process, with data scientists iteratively improving on their models through experimentation.

SR aligns very well with this approach. User-defined constraints and primitive functions allow for exploration of a space of human-readable models belonging to different function classes. One may consider coupling SR with a co-design mechanism for human involvement during the iterative learning process^16^. Domain experts can review each equation and gauge model performance.

### Data efficiency

SR has demonstrated good performance on small datasets. Wilstrup and Kasak found that SR outperformed classical regression and tree-based models across 240 datasets of 250 points or less^26^. This may make SR an appealing option when data is not readily available or is difficult to collect. For example, SR has been successfully applied to modeling electrical flow based on power, voltage, and current with only a few hundred samples^27^. However, such applications typically involve only a few underlying principles or laws, so applicability to other complex systems and relationships remains to be seen.

### Extrapolation

SR models have the potential to extrapolate^8^. This addresses an often-overlooked aspect of ML: the ability to predict outside of the range of the training data. Most contemporary ML models are incapable of this task if the model exhibits complex behavior outside of this range (with the only exceptions among commonly used models being Gaussian processes with a well-chosen kernel^28^ and neural networks with non-standard activation functions^29^). In fact, many SR models are tested by their ability to predict unobserved patterns in the data outside of a training set, in addition to a train-test split on a subset of the data^17,30,31^.

**Fig. 1:**
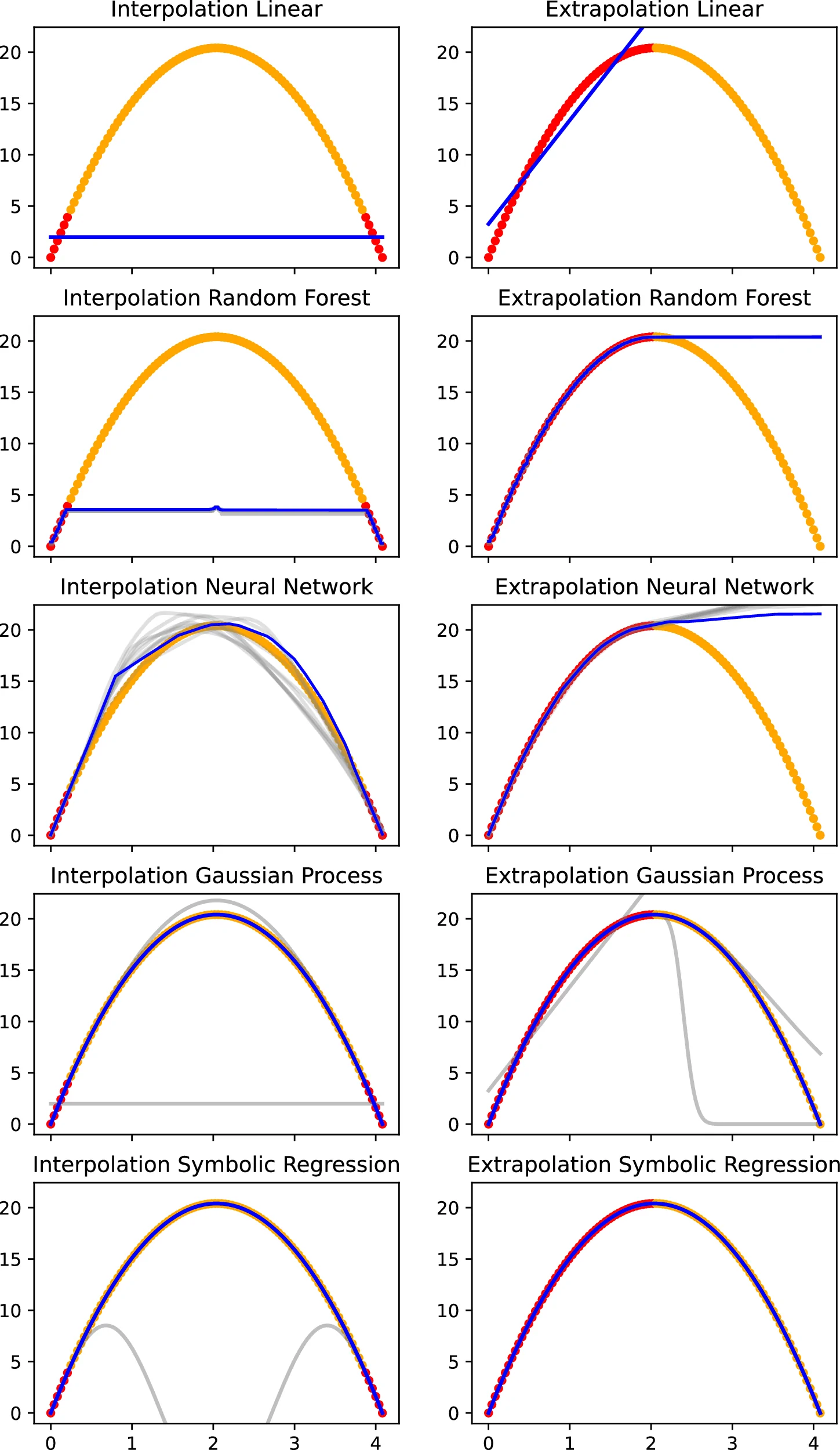
Interpolation and extrapolation potential of model types when learning the trajectory of a thrown ball. The red points represent the training data, and the yellow points testing data. The blue lines represent the predictions of the best-performing model of the type displayed. For classical ML models, the grey lines represent non-optimal solutions learned using different sets of hyperparameters. For SR, only one set of hyperparameters were used, which contained a wide variety of functions (e.g., arithmetic operations, sine, and exponentials) and the grey lines represent the other functions in the Pareto front, excluding those with very high training loss due to insufficient complexity (e.g., a constant). This example was inspired by https://mathematica.stackexchange.com/questions/251180/symbolic-regression and the SR models were trained with PySR.

To illustrate, we conducted an experiment comparing the interpolation and extrapolation capabilities of classical ML models with SR (Fig. 1). Consider a dataset describing a ball thrown into the air (variables include ball height and time). This will represent a parabola when plotting height vs. time (red points are training data, yellow points are testing data). When considering interpolation, we assume training data only includes information during the beginning and end times, and prediction is needed in between. For extrapolation, only the first half of the times are available for training, and prediction is needed for beyond.

Five different modeling approaches are applied (blue lines represent predictions from best performing models, and grey lines represent non-optimal solutions while varying hyperparameters). The linear and random forest models demonstrate a clear misfitting of the testing data, both interpolation and extrapolation. A simple neural network fits testing data reasonably well for interpolation, but poorly for extrapolation. The Gaussian (stochastic) process demonstrates good fitting of testing data in both interpolation and extrapolation, but its performance varies significantly depending on the initial choice of kernel.

For the SR model, only one set of hyperparameters was used, containing a wide variety of functions (e.g., arithmetic operations, sine, and exponentials). The grey lines include functions in the Pareto front, excluding those with very high training loss due to insufficient complexity (e.g., simply containing a constant). As shown in Fig. 1, SR interpolates and extrapolates well to test data. Additionally, SR contained the function used to generate data in the Pareto front (representing the best model for each complexity). Although the example is mathematically simple, this illustrates SR’s potential for finding solutions for unseen inputs outside the training range of data.

### Ethics, fairness, and bias

Potentially, SR interpretability and transparency with observable equations may allow for easier identification of sources of bias or unfairness. It may be feasible to test how altering features influence model predictions (e.g., evaluating different sub-populations based on race/ethnicity or zip code, use of different loss functions) to detect and mitigate bias. Furthermore, sensitive features may be excluded from equations altogether if desired for exploration. Ultimately, non-technical experts (including those involved in adopting models in healthcare) may better understand explicit model expressions to make ethical decisions. Finally, the flexibility of SR may allow for ethical AI considerations to be incorporated directly into the training process.

Multi-objective symbolic regression (MOSR) is a variant SR training algorithm which balances multiple objective functions simultaneously by iteratively constructing the Pareto front of solutions using the multidimensional space of objectives^32^. MOSR models may incorporate fairness metrics directly into its training process by including them as objectives, potentially improving model consistency across groups of interest. Multi-view symbolic regression (MVSR) is a variant SR algorithm that utilizes multiple different data sets simultaneously. MVSR will generate a general parametric model by optimizing parameters across these data sets separately, using the average fitness as the objective^33^. MVSR could be leveraged to consider multiple different patient populations or healthcare systems simultaneously during training to produce a generalized model, potentially reducing bias.

Ultimately, ethical concerns may persist with SR implementation. Simplified SR equations may exhibit complex behaviors, mislead end-users, and inappropriately increase trust^34^. Thus, examination of fairness and bias should be routinely incorporated into SR applications in healthcare, and consideration given to how SR interpretability and transparency may influence adoption and confidence in model implementation.

## What are the limitations of SR?

### Training challenges

SR possesses unique challenges in model training due, in part, to its flexible model structures. Unlike traditional ML models, SR explores an expansive search space of mathematical expressions. This space grows exponentially with the number of input variables, making exhaustive searches computationally infeasible. Furthermore, because the model form evolves during training, gradient-based optimization techniques cannot be easily applied. As a result, SR relies on alternative strategies, such as genetic programming or neural network-based methods, each with their own limitations.

While genetic algorithms are effective at exploring large function spaces, they often propose complex expressions instead of simpler or more interpretable solutions. This can lead to overfitting when noise is present^15^. Penalizing model complexity (loss function) or maintaining a Pareto front may help balance accuracy and simplicity. Still, genetic algorithms may struggle with convergence and sensitivity to hyperparameter settings and random seed values, sometimes yielding very different results.

While NN-powered SR algorithms possess advantages over GP approaches, there are concerns. Inflexibility and reduced interpretability exist among equation learners. The use of gradient descent to tune training weights limits which functions can be used as primitives due to potential discontinuities or singularities. For example, adding division as a primitive function took considerable effort in one experiment^31^. In this case, division units are proposed, with a calculation of a/b, but it is complicated since division creates a pole as b approaches 0 with an abrupt change in convexity. Additionally, because the network still relies on learned linear transformations, expressions will be more complex and lack sparsity, making them less interpretable.

Performance may be diminished among DRL and pre-trained transformer algorithms. While there is promise in benchmark dataset performance, recent independent evaluations indicate that they still lag behind genetic algorithms overall^35,36^. In the case of transformers, there is also the additional issue of poor performance on data that does not bear a resemblance to pre-training data. However, DRL and transformer approaches to SR remain a very active research topic in the community^1,16,30,37,38^.

### Interpretability limitations

While SR models are more interpretable than black-box models, interpretability is context-dependent. Different symbolic expressions can represent the same function, and proving their equivalence often requires manual inspection and mathematical and/or domain expertise. For example, Equation 1 displays two different but equivalent representations of Chebyshev polynomials, a class of functions related to sine and cosine that are important in approximating linear systems and performing polynomial interpolation.

While these two formulas differ in terms of symbolic representation, they still provide the same output for any given input (assuming n is a non-negative integer). To appreciate this, one must understand the complex number definitions of inverse trigonometric functions. Those without a mathematical background might not recognize their equivalence. This symbolic variability can obscure the relationship between a learned model and the real-world system it represents. This supports the need for domain knowledge.

$${T}_{n}\left(x\right)=\left\{\begin{array}{cc}\cos \left(n\,\arccos \left(x\right)\right), \hfill & {\rm{if}}\left|x\right|\le 1, \\ {{\cosh}}\left(n\,{\rm{arcos}}{\rm{h}}\left(x\right)\right), \hfill & {\rm{if}}\,x\ge 1, \\ {\left(-1\right)}^{n}\cosh \left(n\,{\rm{arcos}}{\rm{h}}\left(-x\right)\right), & {\rm{if}}\,x\ge -1.\end{array}\right. \\ {T}_{n}\left(x\right)=\frac{1}{2}\left({\left(x-\sqrt{{x}^{2}-1}\right)}^{n}+{\left(x+\sqrt{{x}^{2}-1}\right)}^{n}\right)\\ =\frac{1}{2}\left({\left(x-\sqrt{{x}^{2}-1}\right)}^{n}+{\left(x-\sqrt{{x}^{2}-1}\right)}^{-n}\right)$$

Equation 1: Two different equivalent representations of Chebyshev polynomials of the first kind.

The first displayed equation is a piecewise trigonometric representation of Chebyshev polynomials of the first kind. The second is a representation involving only square roots and exponents. Despite their symbolic dissimilarity, one can apply complex exponential and logarithmic identities to show that they are equivalent. In cases where the symbolic representation plays a role in model interpretability analysis, missing such equivalences might have significant consequences.

### Extrapolation limitations

While SR may exhibit more complex extrapolation behavior than other model types, its performance may remain poor outside of the range of the training data distribution. This is particularly true when there is a lack of domain-specific knowledge or constraints^2,39^. Additionally, models trained using only basic arithmetic functions may produce high-degree polynomial models which can oscillate or deviate excessively at more extreme values of the input features (e.g., Runge’s phenomenon^40^). Further, extrapolation may be worse due to complex model overfitting, algorithmic variance, or relationships that are difficult to capture with simple expressions. Strategies to address extrapolation concerns may include withholding values in extreme regions for testing, leveraging domain knowledge when choosing primitive functions, and carefully analyzing learned SR equations to understand their behavior.

### Confirmatory analysis

SR is most suitable for exploratory or prediction analyses, rather than confirmatory data analyses. SR is inherently data-driven and is particularly applicable in settings with uncertain or complex relationships where prespecified model structures are not available. Extensions to support confirmatory analyses, including uncertainty quantification and formal inference, may represent areas for future work.

### No method fits all

Lastly, no single analytic method fits all data. In practice, non-exhaustive algorithms are biased in which models they discover in their search space. SR models can miss desirable solutions during the training process. Additionally, SR algorithms, particularly genetic algorithms, are considered high variance, where small changes in training data result in very different solutions^41^. Finding the right constraints or hyperparameters to steer the final model away from undesirable solutions remains a challenge.

SR represents only one of many interpretable methods available (e.g., rule mining and sparse linear regression). One should consider all options when selecting an approach to model building. SR remains scarcely tested in healthcare, and as new applications emerge, we must be wary of “over-optimism.”^42^

## Applying SR to healthcare

In healthcare, non-interpretable models are unlikely to see actual implementation. Doctors and patients alike are very hesitant to trust a model that cannot be understood or analyzed^43,44^. Hence, direct clinical application of mainstream ML models remains restricted. The interpretability of SR offers a means of improving interpretability while maintaining comparable performance to black-box models.

Although SR has seen less use in medicine than other domains, newer and emerging applications have been identified. La Cava et al.^45^ introduce a straightforward approach to applying SR to high-dimensional EHR data using FEAT (Feature Engineering Automation Tool). They trained EHR prediction models that are intuitively interpretable and accurate in the context of hypertension classification. Next, there have been several studies applying MOSR to medicine, recognizing its ability to naturally handle imbalanced data. Arina et al. trained a MOSR model for 1-year post-surgery mortality prediction^46^. This model outperformed classical regression and tree-based models, while not overfitting the training set. Similarly, Ferrari et al. utilized EHR data to build a MOSR model for simultaneously predicting ICU bloodstream infections and antimicrobial resistance^47^. The models demonstrated improved performance in most metrics compared to classical regression and tree-based models as well. Interestingly, they were also the only models to obtain a non-zero specificity other than decision trees. Additionally, the MOSR models were compact and simple, consisting of only 3–6 features and a few terms (with one exception), with logarithms, powers, square roots, and basic arithmetic operations as the only functions involved.

**Fig. 2:**
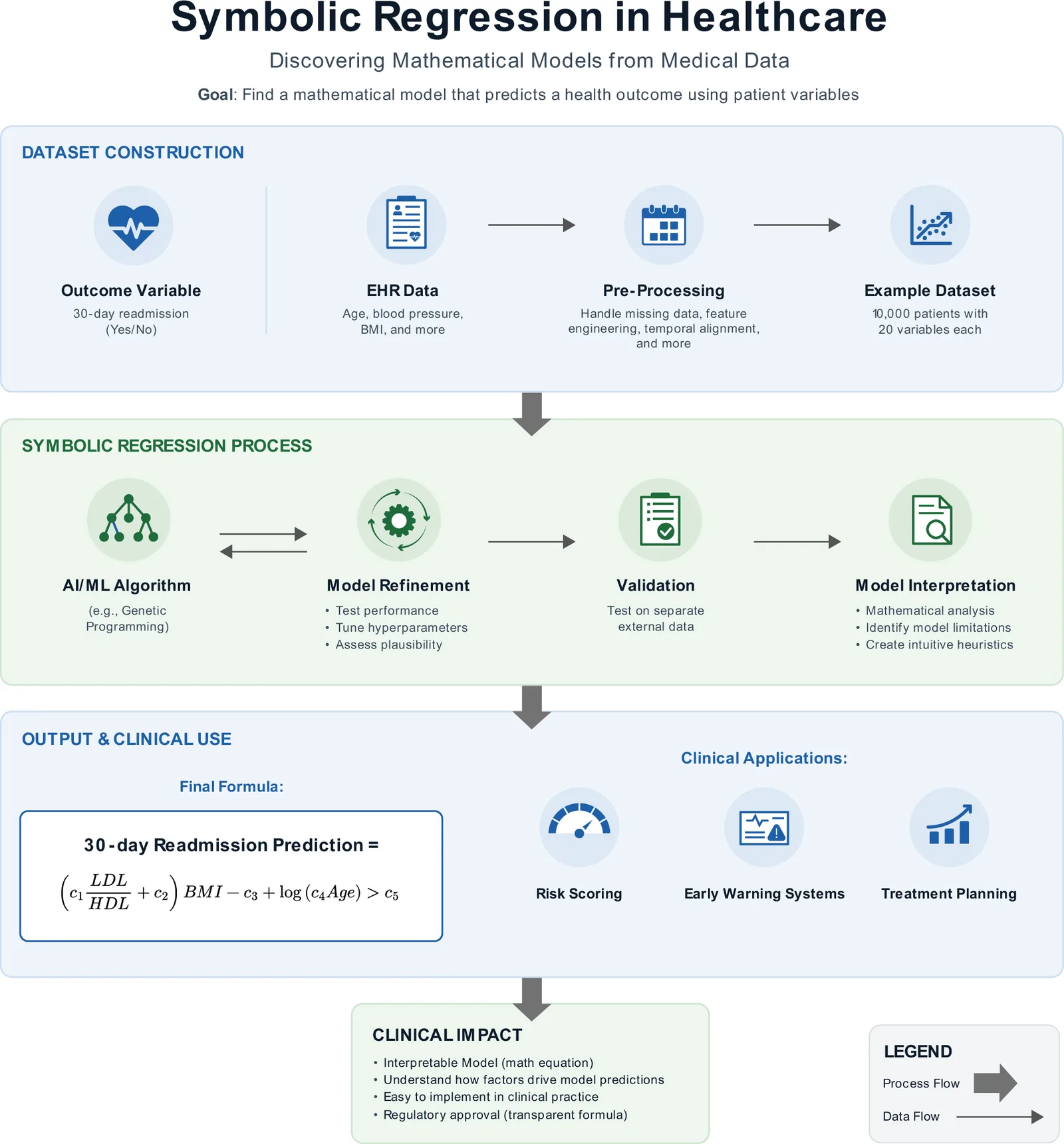
Schematic Example of Clinical SR Model Building and Deployment Process. This schematic is divided into three major steps, with wide arrows representing process fl ow and narrow arrowrepresenting data fl ow. *Dataset construction:* Create a dataset by selecting a clinically relevant outcome variable,extracting related features from electronic health records (EHR), and pre-processing the data (e.g., handling missingvalues and standardizing values). Symbolic regression process: Apply an SR algorithm (e.g., genetic programming) tolearn candidate mathematical expressions from the data, refi ne the resulting models through iterative testing,tuning, and performance assessment, validate the fi nal equations on an external dataset to evaluate generalizability,and provide clinical interpretation of the derived equations (identify limitations and do a “sanity check”). Output & clinical use: The fi nal output is an SR formula (e.g., 30-day hospital readmission prediction) that may be clinicallyinterpretable and deployed in applications such as calculating patient risk scores, powering alert systems embeddedin EHR platforms, and informing personalized treatment decisions.

Figure 2 provides a schematic example of how one might utilize SR for prediction modeling using electronic health record (EHR) data. Patient variables are selected (e.g., age, blood pressure) to predict a pre-determined outcome (e.g., hospital readmission). Pre-processing may be required (e.g., temporal alignment, summary variable creation, data normalization, and missing data handling), which will likely influence interpretability and transparency of the final SR expression. Clinical insights (a priori knowledge) may also be applied through feature selection and engineering (e.g., comorbidity score, or building block of BUN/creatinine ratio). After this stage, data may be split for testing, training, and internal validation.

Next is the SR process, typically through genetic algorithm or neural network-based approaches. SR packages incorporating an ML/AI algorithm search for an optimal equation according to a user provided or pre-defined objective (typically a balance between loss and complexity). Learned equation(s) are then assessed according to their performance, form, and plausibility. Depending on this assessment, model refinement may occur, primarily consisting of tuning hyperparameters through trial and error and/or adjusting constraints. Once models have been evaluated on internal test sets and a final candidate model(s) are selected, their performance(s) will be verified using external data sets. If verification is successful, a final model is selected and interpreted, including a final “sanity check” (e.g., *do the formula relationships make sense?*).

Additional analyses (e.g., statistical testing^48^, differential sensitivity analysis^49^, and equation simplification^50^) can help better understand selected model properties and limitations. Statistical verification of unexpected terms in the model^45^ may lead to the discovery of previously unknown relationships. With consideration of bias, such analyses may identify patient groups that the model might perform poorly on. Depending on the complexity of the model, one may also develop intuitive heuristics, provable rules, and visual aids describing the model’s behavior. Explainable AI tools may be utilized (but not solely relied on) during this process to generate feature importance scores and facilitate comparison with black-box models trained for the same task. In particular, the relationship among important features identified by SR may provide more insights that are clinically significant.

Finally, the resulting formula and its potential clinical use are considered. The formula is presented as an explicit mathematical function, typically including numerical coefficients and clearly defined variable names corresponding to clinical features. This transparency allows domain experts, general users, and regulatory bodies to more easily conduct their own evaluations of the model structure and output. Clinical applications may involve risk scoring (for classification models), development of early warning systems (e.g., EHR alerts for patients at high risk of rehospitalization), and treatment planning. Prior to deployment, a number of requirements remain, including regulatory requirements, health system approval and clinician buy-in (perhaps improved if more interpretable), bias and fairness evaluation, and ongoing monitoring of performance.

While SR may apply to both diagnostic and prognostic classification tasks, continuous risk prediction models possess unique consideration. Prediction with SR models may provide a concise and interpretable means of estimating the risk of future events (e.g., based on electronic health record data). Expert clinicians may review SR equations and consider alternative factors and relationships that are not found in current guidelines or meta-analyses. To achieve these interpretability benefits, attention must be given to the pre-processing of input data (as with other ML models that offer explainability). Models developed from summarized patient-level features of temporal data may omit important variations within SR expressions (as opposed to raw features in the original clinical context)^45^.

Future applications of SR in medicine may leverage the benefits described above. Better drug absorption or organ function models may be developed using symbolic forms. For example, estimated kidney function may include the calculation of glomerular filtration rate (GFR) based on age, weight, creatinine, and sex (Cockcroft-Gault Formula). While a simple equation may easily be used for medication dosing, it is less accurate with worse kidney function and not broadly applicable to different subpopulations. However, a genetic SR equation applied to kidney transplant patients has shown high accuracy and improved bias at lower GFR^51^. SR may also help tailor equations to other specific populations (i.e., younger children). Future approaches may similarly apply novel methods, such as constrained genetic perturbation, taking existing GFR equations and modifying them to achieve better performance^52^.

Furthermore, SR may describe mechanisms of how behavior and lifestyle influence health with greater transparency. For example, a SR model could provide a function that delineates how diet, physical activity, and medication influence blood glucose in diabetes. One approach involved modeling blood glucose, with customized equations involving carbohydrate intake and insulin use by example simulated patients^53^. Its strong performance also reflected situations of hyper and hypoglycemia, which possess clinical value for a given individual. Future SR applications involving human patients with diabetes, incorporating objective data (e.g., wearable steps for physical activity and continuous glucose monitoring), will offer more interpretable expressions that relate how real-world behaviors affect human physiology.

Successful healthcare implementation of SR models will need to consider additional issues. Data leakage arises when models are trained on inappropriate or hidden features that are directly related to a prediction outcome (for example, medical record IDs shared within a common hospital in a multicenter study^54^). Thus, it is important to carefully consider mechanisms and relationships contained within SR equations, including the plausibility and relevance of all features. Next, a covariate or distribution shift occurs when there are distributional differences across populations or healthcare organizations^55^. This may also reflect variations in demographics, clinical practices, and data collection methods. SR models exhibiting strong interpolation and extrapolation behavior may prove more resilient against covariate shifts than most types of models. However, strategies to continually update an SR model as data shifts occur will still be important for consistently maintaining performance across healthcare organizations^56^.

Generally, the regulation and validation of AI models are increasingly recognized challenges for healthcare implementation^57^. SR models would similarly require the same level of evaluation and monitoring throughout the development lifecycle as non-SR models. Greater interpretability of SR models may be advantageous, but model expression complexity may raise concerns upon expert review scrutiny. Existing AI assurance laboratories or localized validation may help lead to future adoption of prediction models, but ongoing monitoring with SR expression revisions will likely be necessary (a similar need exists for all ML models^58^). In contrast, diagnostic model (e.g., imaging analysis) implementation may be even more challenging, being perceived as having greater medical and legal risk. Overall, these issues require attention for real-world clinical deployment, with the realization that SR may introduce benefits as well as unique challenges.

## Conclusion

SR addresses the concern of machine learning model interpretability. Although its performance can vary, it offers the potential for better performance and generalizability, particularly when the data size is small. SR may be considered in healthcare data modeling, recognizing its limitations and the need for domain knowledge.

Unlike “black box” ML, SR generates transparent, human-readable equations. This is invaluable in clinical settings, where clinicians can use equations to interpret risk and make informed decisions. SR may offer insights on mechanistic relationships, such as between lifestyle and physiology. Health systems may consider the adoption of externally verified SR models utilizing EHR data, given the ubiquitous concerns of trust and the opaqueness of current, difficult-to-interpret algorithms.

## Supplementary information

Transparent Peer Review File
