# Supplementary material for: Introduction to using symbolic regression for interpretable machine learning in healthcare
Source: Commun Med (Lond). 2026 Oct 9;6:524. doi: 10.1038/s43856-026-01914-x (PMC13652824; doi:10.1038/s43856-026-01914-x)
Supplement: Supplementary file 1 [file 43856_2026_1914_MOESM1_ESM.pdf]

nature portfolio

Peer Review File

Introduction to Using Symbolic Regression for Interpretable Machine Learning in Healthcare

Corresponding Author: Dr Michael Ferguson

This file contains all reviewer reports in order by version, followed by all author rebuttals in order by version.

Version 0:

Reviewer comments:

Reviewer #1

(Remarks to the Author)
1. The commentary presents a clear, well-structured overview of symbolic regression (SR) with broad, up-to-date coverage, including a strong linkage to healthcare and interpretability motivation.

2. The extrapolation advantage of SR is presented as near-general. However, in practice, the success of extrapolation is highly problem-dependent and not guaranteed.

3. The phrase, "symbolic regression," does not appear anywhere in the reference cited as 22, "Bahri et al., Explaining Neural Scaling Laws." The claim, "Bahri et al found that SR outperformed classical regression and tree-based models across 240 datasets of 250 points or less," raises serious credibility issues.

4. The manuscript alternates between portraying SR as “easy to use” and later emphasizing its sensitivity to hyperparameters, randomness, and domain expertise, raising consistency issues.

5. Ethical, fairness, and bias considerations specific to SR in healthcare are not sufficiently addressed.

6. Methodological and implementation details for Figure 1 are not sufficient enough to interpret it rigorously.

7. Medical examples are promising but largely anecdotal. Limitations around data leakage, covariate shift, regulatory validation, and clinical deployment are not sufficiently discussed.

Reviewer #2

(Remarks to the Author)
This manuscript presents a commentary on symbolic regression as an approach for interpretable machine learning or statistical modelling in healthcare. I generally enjoyed reading the paper and find the topic timely and relevant, given the ongoing discussion about transparency and interpretability of prediction models in clinical applications.
That said, I am not entirely sure who the intended audience of this commentary is. While the paper avoids technical detail and is written in an accessible style, I do not think it provides enough conceptual explanation for readers without a strong methodological background. Many key ideas are mentioned rather briefly, and important assumptions and distinctions remain implicit. As a result, non-expert readers may struggle to fully understand what symbolic regression can realistically offer in practice, while more experienced readers may find the discussion somewhat superficial. Clarifying the target audience and adapting the level of explanation accordingly would, in my view, strengthen the manuscript.
- Related to this, it would be helpful if the authors were more explicit about the types of problems they have in mind. In particular, it remains unclear whether the focus is primarily on diagnostic models, prognostic models, or both. This distinction is important in healthcare, as the role of interpretability, the requirements for validation, and the consequences of model use differ substantially between diagnostic and prognostic settings.
- I also found it somewhat unclear whether symbolic regression is mainly intended as a tool for prediction and exploratory analysis, or whether the authors see a role for it in more confirmatory analyses. Readers with a statistical background may wonder how symbolic regression fits into settings where prespecified models, uncertainty quantification, and formal inference are central. Some clarification on this point would be useful.

- The commentary would, in my opinion, benefit greatly from at least one worked example. Even a simple, schematic example could help readers understand how symbolic regression works in practice, what the resulting models look like, and how interpretability is achieved compared to more standard machine learning approaches or structured additive models (e.g., GAMs) from statistics.
- In addition, I think the manuscript could engage more explicitly with the fact that, in many real-world prediction problems, a large number of different models often achieve very similar predictive performance. This point has been discussed prominently by Cynthia Rudin and others, for example in the context of the Rashomon set. From this perspective, symbolic regression should perhaps be positioned more clearly as one promising option within a broader set of similarly performing models, rather than implicitly as a method that resolves most existing limitations.
- More generally, at several points the commentary reads as if symbolic regression might be a broadly superior solution to current challenges in interpretable machine learning and statistical modelling. I would encourage the authors to soften this impression. As emphasized repeatedly in the methodological literature, there is no “one method fits all” solution. This has been articulated clearly, for example, by Strobl and Leisch (“Against the ‘one method fits all data sets’ philosophy for comparison studies in methodological research”, DOI: 10.1002/bimj.202200104) as well as by Anne-Laure Boulesteix and colleagues. Making this perspective more explicit would help to present a more balanced and realistic view.

Reviewer #3

(Remarks to the Author)
This is a clear and well written commentary on symbolic regression as an interpretable machine learning technique and its potential application in healthcare. The authors do a good job explaining the topic for readers with a medical background and convincingly argue the strengths of symbolic regression with illustrative examples.

The manuscript references examples of symbolic regression being successfully used in mathematically oriented fields such as physics, engineering, economics and climate research. The authors persuasively argue that certain characteristics of symbolic regression would be beneficial in healthcare, such as interpretability. The authors also argue that knowledge of symbolic regression application to healthcare is limited.

However, its applicability in healthcare is made less clear. The main argument the authors give that symbolic regression is applicable to medicine is twofold: (1) Medical datasets are used as benchmarks for symbolic regression algorithms and (2) examples from recent literature of successful application of symbolic regression. It begs the question: If symbolic regression is already being successfully applied in healthcare, would a review of those methods be more appropriate? What is the novelty of this manuscript?

The examples of symbolic regression (e.g. interpolation and extrapolation, figure 1) are didactic, but mathematically trivial. It would really help the stated purpose of the manuscript if examples taken were from medicine.

The authors frame symbolic regression as a method for finding the "true or approximate functional form of the ground truth". They do not clearly explain what this means in a medical context. It is also unclear from the text if symbolic regression is being presented as a modeling technique (where the task is to find a mathematical representation which matches observations well enough for some purpose) or discovering a previously unknown true underlying functionality.

Finally, on framing: The manuscript is described as a commentary, but reads more like a tutorial on symbolic regression.

Version 1:

Reviewer comments:

Reviewer #1

(Remarks to the Author)
Thanks for addressing the comments but the credibility concerns remain. For instance, "Bahri et al" now became “Wilstrup and Kasak" and the change is not highlighted in the bibliography.

Lines 568 - 571 includes run-off text: "La Cava et al. conduct the first study utilizing SR for EHR prediction modeling and demonstrate the viability of their approach by performing a comprehensive analysis and validation of their methodology and models."

Similarly, lines 540-542 contain "Aldeia et al. assess a wide range of state-of-the-art SR algorithms using modified benchmark problems, providing an objective overview of their current performances."

Reviewer #2

(Remarks to the Author)
I thank the authors for the careful revision of the manuscript and the detailed answers to my comments. I have the feeling that the target group of the comment is clearer now and it reads more realistic regarding the methods advantages.

However, I have one remaining follow-up point:

The author correctly acknowledge in their answer that symbolic regression is most suitable for exploratory or prediction analyses but not for confirmatory data analyses. However, I did not find that statement in the manuscript. I think it would be good for the reader if this is also acknowledged there - please forgive me if I simply overlooked this.

Open Access This Peer Review File is licensed under a Creative Commons Attribution 4.0 International License, which permits use, sharing, adaptation, distribution and reproduction in any medium or format, as long as you give appropriate credit to the original author(s) and the source, provide a link to the Creative Commons license, and indicate if changes were made.
In cases where reviewers are anonymous, credit should be given to 'Anonymous Referee' and the source.
The images or other third party material in this Peer Review File are included in the article's Creative Commons license, unless indicated otherwise in a credit line to the material. If material is not included in the article's Creative Commons license and your intended use is not permitted by statutory regulation or exceeds the permitted use, you will need to obtain permission directly from the copyright holder.
To view a copy of this license, visit https://creativecommons.org/licenses/by/4.0/

May 22, 2026

RE: Manuscript #: COMMSMED-25-3026

Dear Referees,

Thank you for the thoughtful and constructive reviews of our manuscript "**Symbolic Regression for Interpretable Machine Learning in Healthcare: A Commentary**". We appreciate your careful reading and insightful suggestions. In response, we have revised the manuscript to clarify its intended audience and scope, soften claims that could be interpreted as overstating the advantages of symbolic regression (SR), correct the citation error identified by Referee 1, strengthen the discussion of fairness and implementation, and add a new healthcare-oriented schematic example. We have also made a number of revisions to improve precision, consistency, and readability throughout the manuscript.

<u>Referee #1:</u>

1. The extrapolation advantage of SR is presented as near-general. However, in practice, the success of extrapolation is highly problem-dependent and not guaranteed.

We agree that extrapolation capabilities of SR models may vary. While SR has the potential for more complex extrapolation behavior than most other algorithms, this does not necessarily imply consistently strong extrapolation performance. We have added text (lines 278-287) discussing SR's limitations with extrapolation tasks in further detail. First, we note the importance of domain specific knowledge and constraints in extrapolation. Next, we call attention to the unreliable extrapolation behaviors of SR models that only use basic arithmetic functions. Finally, we provide some common sources of SR model extrapolation error (e.g. algorithmic variance) and present mitigate strategies (e.g. withholding values in extreme regions for testing).

> "While SR may exhibit more complex extrapolation behavior than other model types, its performance may remain poor outside of the range of the training data distribution. This is particularly true when there is a lack of domain-specific knowledge or constraints[2,39]. Additionally, models trained using only basic arithmetic functions may produce high-degree polynomial models which can oscillate or deviate excessively at more extreme values of the input features (e.g. Runge's phenomenon[40]). Further, extrapolation may be worse due to complex model overfitting, algorithmic variance, or relationships that are difficult to capture with simple expressions. Strategies to address extrapolation concerns may include withholding values in extreme regions for testing, leveraging domain knowledge when choosing primitive functions, and carefully analyzing learned SR equations to understand their behavior."

2. The phrase, "symbolic regression," does not appear anywhere in the reference cited as 22, "Bahri et al., Explaining Neural Scaling Laws." The claim, "Bahri et al found that SR outperformed classical regression and tree-based models across 240 datasets of 250 points or less," raises serious credibility issues.

We thank the referee for pointing out this error. We have corrected the citation and verified all references for accuracy.

Citation 26 now includes the appropriate source: "Symbolic regression outperforms other models for small data sets".

> "Wilstrup and Kasak found that SR outperformed classical regression and tree-based models across 240 datasets of 250 points or less[26]."

3. The manuscript alternates between portraying SR as "easy to use" and later emphasizing its sensitivity to hyperparameters, randomness, and domain expertise, raising consistency issues.

We agree that the brief section on "Ease of Use" may be misleading, and conflicts with the other challenges and concerns described. To address this, we redefined this section as "Practical Application" (lines 127-135) to describe how SR models may be developed and trained with few lines of code through available software packages.

> "***Practical Application***
>
> Several software packages are available for SR implementation which provide a streamlined user focused experience. For genetic algorithms, PySR[13] offers a highly flexible and customizable open-source Python tool with a robust API that provides tuning and workflow tips for users (the backend uses a highly-optimized Julia library). For Neural Network algorithms, Deep Symbolic Optimization (DSO) offers an actively developed unified framework for deep symbolic learning (including SR)[20] and provides an sklearn-style interface for ease of use. SR models utilizing such packages may be trained with only a few lines of code, with the ability to review candidate equations during fitting as well as select a specific expression for prediction."

In addition, we moved the text on how SR does not require *a priori* knowledge about the data to the section on "Integration of Domain Knowledge" (lines 136-147).

> **"*Integration of Domain Knowledge***
>
> Despite its mathematical nature, SR does not require *a priori* knowledge about the data (though Bayesian approaches can be taken)[19]. While domain expertise and mathematical

knowledge offer advantages, SR is an entirely data-driven process like other ML models. Hence, it requires no specialized knowledge to use.

Alternatively, SR can use domain knowledge. Usually, derived model functions only include pre-defined specific primitive functions. Therefore, by utilizing domain knowledge, one can build models including only the most relevant and meaningful primitives. For example, when studying growth dynamics, generalized logistic models known as hyperbolastic models are often used[21]. By including inverse hyperbolic sine, the exponential function, square, square root, and hyperbolic tangents into the set of primitive functions, the search space (set of possible equations discoverable during training) will include relevant hyperbolastic models and similar variants."

4. Ethical, fairness, and bias considerations specific to SR in healthcare are not sufficiently addressed.

We agree that ethics, fairness, and bias in machine learning models are critical. While empirical studies specially focused on SR in healthcare are limited, we have expanded the discussion on lines 212-236 to situate SR within the broader literature on fairness in machine learning and to highlight both opportunities and risks.

"*Ethics, Fairness, and Bias*

Potentially, SR interpretability and transparency with observable equations may allow for easier identification of sources of bias or unfairness. It may be feasible to test how altering features influence model predictions (e.g. evaluating different sub-populations based on race/ethnicity or zip code, use of different loss functions) to detect and mitigate bias. Furthermore, sensitive features may be excluded from equations altogether if desired for exploration. Ultimately, non-technical experts (including those involved in adopting models in healthcare) may better understand explicit model expressions to make ethical decisions. Finally, the flexibility of SR may allow for ethical AI considerations to be incorporated directly into the training process.

Multi-Objective Symbolic Regression (MOSR) is a variant SR training algorithm which balances multiple objective functions simultaneously by iteratively constructing the Pareto front of solutions using the multidimensional space of objectives[32]. MOSR models may incorporate fairness metrics directly into its training process by including them as objectives, potentially improving model consistency across groups of interest. Multi-View Symbolic Regression (MVSR) is a variant SR algorithm that utilizes multiple different data sets simultaneously. MVSR will generate a general parametric model by optimizing parameters across these data sets separately, using the average fitness as the objective[33]. MVSR could be leveraged to consider multiple different patient populations

or healthcare systems simultaneously during training to produce a generalized model, potentially reducing bias.

Ultimately, ethical concerns may persist with SR implementation. Simplified SR equations may exhibit complex behaviors, mislead end-users, and inappropriately increase trust[34]. Thus, examination of fairness and bias should be routinely incorporated into SR applications in healthcare, and consideration given to how SR interpretability and transparency may influence adoption and confidence in model implementation."

5. Methodological and implementation details for Figure 1 are not sufficient enough to interpret it rigorously.

We now provide more details on the data, methods, and implementation to describe interpolation/extrapolation potential across models in Figure 1 (lines 190-210).

"To illustrate, we conducted an experiment comparing the interpolation and extrapolation capabilities of classical ML models with SR (Figure 1)[59]. Consider a dataset describing a ball thrown into the air (variables include ball height and time). This will represent a parabola when plotting height vs. time (red points are training data, yellow points are testing data). When considering interpolation, we assume training data only includes information during the beginning and end times, and prediction is needed in-between. For extrapolation, only the first half of times are available for training, and prediction is needed for beyond.

Five different modeling approaches are applied (blue lines represent predictions from best performing models, and grey lines represent non-optimal solutions while varying hyperparameters). The linear and random forest models demonstrate a clear misfitting of the testing data, both interpolation and extrapolation. A simple neural network fits testing data reasonably well for interpolation, but poorly for extrapolation. The Gaussian (stochastic) process demonstrates good fitting of testing data in both interpolation and extrapolation, but its performance varies significantly depending on the initial choice of kernel.

For the SR model, only one set of hyperparameters was used, containing a wide variety of functions (e.g. arithmetic operations, sine, and exponentials). The grey lines include functions in the Pareto front, excluding those with very high training loss due to insufficient complexity (e.g., simply containing a constant). As shown in Figure 1, SR interpolates and extrapolates well to test data. Additionally, SR contained the function used to generate data in the Pareto front (representing the best model for each complexity). Although the example is mathematically simple, this illustrates SR's potential for finding solutions for unseen inputs outside the training range of data."

6. Medical examples are promising but largely anecdotal. Limitations around data leakage, covariate shift, regulatory validation, and clinical deployment are not sufficiently discussed.

We agree that examples of SR in medicine are limited and anecdotal. We added to lines 385-407 of our section on "Applying SR to Healthcare" additional text that briefly discusses data leakage, covariate shift, regulatory validation, and clinical deployment.

> "Successful healthcare implementation of SR models will need to consider additional issues. Data leakage arises when models are trained on inappropriate or hidden features that are directly related to a prediction outcome (for example, medical record IDs shared within a common hospital in a multicenter study[54]). Thus, it is important to carefully consider mechanisms and relationships contained within SR equations, including the plausibility and relevance of all features. Next, covariate or distribution shift occurs when there are distributional differences across populations or healthcare organizations[55]. This may also reflect variations in demographics, clinical practices, and data collection methods. SR models exhibiting strong interpolation and extrapolation behavior may prove more resilient against covariate shifts than most types of models. However, strategies to continually update an SR model as data shifts occur will still be important for consistently maintaining performance across healthcare organizations[56].
>
> Generally, regulation and validation of AI models are increasingly recognized challenges for healthcare implementation[57]. SR models would similarly require the same level of evaluation and monitoring throughout the development lifecycle as non-SR models. Greater interpretability of SR models may be advantageous, but model expression complexity may raise concerns upon expert review scrutiny. Existing AI assurance laboratories or localized validation may help lead to future adoption of prediction models, but ongoing monitoring with SR expression revisions will likely be necessary (a similar need exists for all ML models[58]). In contrast, diagnostic model (e.g., imaging analysis) implementation may be even more challenging, being perceived as having greater medical and legal risk. Overall, these issues require attention for real-world clinical deployment, with the realization that SR may introduce benefits as well as unique challenges."

Referee #2 (Remarks to the Author):

1. … I am not entirely sure who the intended audience of this commentary is. While the paper avoids technical detail and is written in an accessible style, I do not think it provides enough conceptual explanation for readers without a strong methodological background. Many key ideas are mentioned rather briefly, and important assumptions and distinctions remain implicit. As a result, non-expert readers may struggle to fully understand what

symbolic regression can realistically offer in practice, while more experienced readers may find the discussion somewhat superficial. Clarifying the target audience and adapting the level of explanation accordingly would, in my view, strengthen the manuscript.

The intended audience as clinician researchers/scientists, translational researchers, medical informaticists, and populations health researchers, who are familiar with the challenges of implementing AI in healthcare but may not have prior exposure to symbolic regression. To better align with this audience, we have added additional text regarding implementation with the following (in relation to other referee concerns):

- A schematic example of an SR model building pipeline (see Referee 2, #4)
- A discussion concerning challenges that must be addressed by futures studies seeking to create SR models for deployment in a healthcare setting (see Referee 1, #6).
- A discussion concerning how SR might interact with ethics, fairness, and bias considerations when applying it to medicine (see Referee 1, #4).
- Further explanation of Figure 1 to ensure the reader can better understand extrapolation advantages with SR in principle (see Referee 1, #5).

2. Related to this, it would be helpful if the authors were more explicit about the types of problems they have in mind. In particular, it remains unclear whether the focus is primarily on diagnostic models, prognostic models, or both. This distinction is important in healthcare, as the role of interpretability, the requirements for validation, and the consequences of model use differ substantially between diagnostic and prognostic settings.

We would consider the application of SR to be relevant to both diagnostic and prognostic models.

- We provide additional text related to validation and evaluation in the context of diagnostic and prognostic models (see Referee 1, #6).
- We call attention to predictive models that incorporate broad electronic health record data and include an additional schematic example (new Figure 2).
- We provide additional text (lines 357-365) discussing the unique factors that must be considered for SR models of continuous risk prediction versus classification:

  "While SR may apply to both diagnostic and prognostic classification tasks, continuous risk prediction models possess unique consideration. Prediction with SR models may provide a concise and interpretable means of estimating the risk of future events (e.g., based on electronic health record data). Expert clinicians may review SR equations and consider alternative factors and relationships that are not found in current guidelines or meta-analyses. To achieve these interpretability benefits, attention must be given to the

pre-processing of input data (as with other ML models that offer explainability). Models developed from summarized patient-level features of temporal data may omit important variations within SR expressions (as opposed to raw features in the original clinical context)[45]."

3. I also found it somewhat unclear whether symbolic regression is mainly intended as a tool for prediction and exploratory analysis, or whether the authors see a role for it in more confirmatory analyses. Readers with a statistical background may wonder how symbolic regression fits into settings where prespecified models, uncertainty quantification, and formal inference are central. Some clarification on this point would be useful.

We position SR primarily as a tool for prediction and exploratory modeling rather than confirmatory analysis. SR is inherently data-driven and is particularly suited to settings with uncertain or complex relationships where prespecified model structures are not available. We acknowledge that extensions to support confirmatory analyses, including uncertainty quantification and formal inference are important areas for future work. While such approaches may be feasible, they remain relatively underdeveloped in the current literature and are beyond the scope of this introductory manuscript.

4. The commentary would, in my opinion, benefit greatly from at least one worked example. Even a simple, schematic example could help readers understand how symbolic regression works in practice, what the resulting models look like, and how interpretability is achieved compared to more standard machine learning approaches or structured additive models (e.g., GAMs) from statistics.

We agree that a detailed example would be helpful and added a schematic (Figure 2) as well as explanatory text (lines 319-356). This schematic demonstrates the steps involved in SR development, evaluation, interpretation, and implementation. Additionally, it shows how an SR model might appear visually.

> "Figure 2 provides a schematic example of how one might utilize SR for prediction modeling using electronic health record (EHR) data. Patient variables are selected (e.g., age, blood pressure) to predict a pre-determined outcome (e.g., hospital readmission). Pre-processing may be required (e.g., temporal alignment, summary variable creation, data normalization and missing data handling), which will likely influence interpretability and transparency of the final SR expression. Clinical insights (*a priori* knowledge) may also be applied through feature selection and engineering (e.g., comorbidity score, or building block of BUN/creatinine ratio). After this stage, data may be split for testing, training, and internal validation.

Next is the symbolic regression process, typically through genetic algorithm or neural network-based approaches. SR packages incorporating an ML/AI algorithm search for an optimal equation according to a user provided or pre-defined objective (typically a balance between loss and complexity). Learned equation(s) are then assessed according to their performance, form, and plausibility. Depending on this assessment, model refinement may occur, primarily consisting of tuning hyperparameters through trial and error and/or adjusting constraints. Once models have been evaluated on internal test sets and a final candidate model(s) are selected, their performance(s) will be verified using external data sets. If verification is successful a final model is selected and interpreted, including a final "sanity check" (e.g., *do the formula relationships make sense?*).

Additional analyses (e.g. statistical testing[48], differential sensitivity analysis[49], and equation simplification[50]) can help better understand selected model properties and limitations. Statistical verification of unexpected terms in the model[45] may lead to the discovery of previously unknown relationships. With consideration of bias, such analyses may identify patient groups that the model might perform poorly on. Depending on the complexity of the model, one may also develop intuitive heuristics, provable rules, and visual aids describing the model's behavior. Explainable AI tools may be utilized (but not solely relied on) during this process to generate feature importance scores and facilitate comparison with black-box models trained for the same task. In particular, the relationship among important features identified by symbolic regression may provide more insights that are clinically significant.

Finaly, the resulting formula and its potential clinical use are considered. The formula is presented as an explicit mathematical function, typically including numerical coefficients and clearly defined variable names corresponding to clinical features. This transparency allows domain experts, general users, and regulatory bodies to more easily conduct their own evaluations of the model structure and output.. Clinical applications may involve risk scoring (for classification models), development of early warning systems (e.g., EHR alerts for patients at high risk of rehospitalization), and treatment planning. Prior to deployment, a number of requirements remain, including regulatory requirements, health system approval and clinician buy-in (perhaps improved if more interpretable), bias and fairness evaluation, and ongoing monitoring of performance."

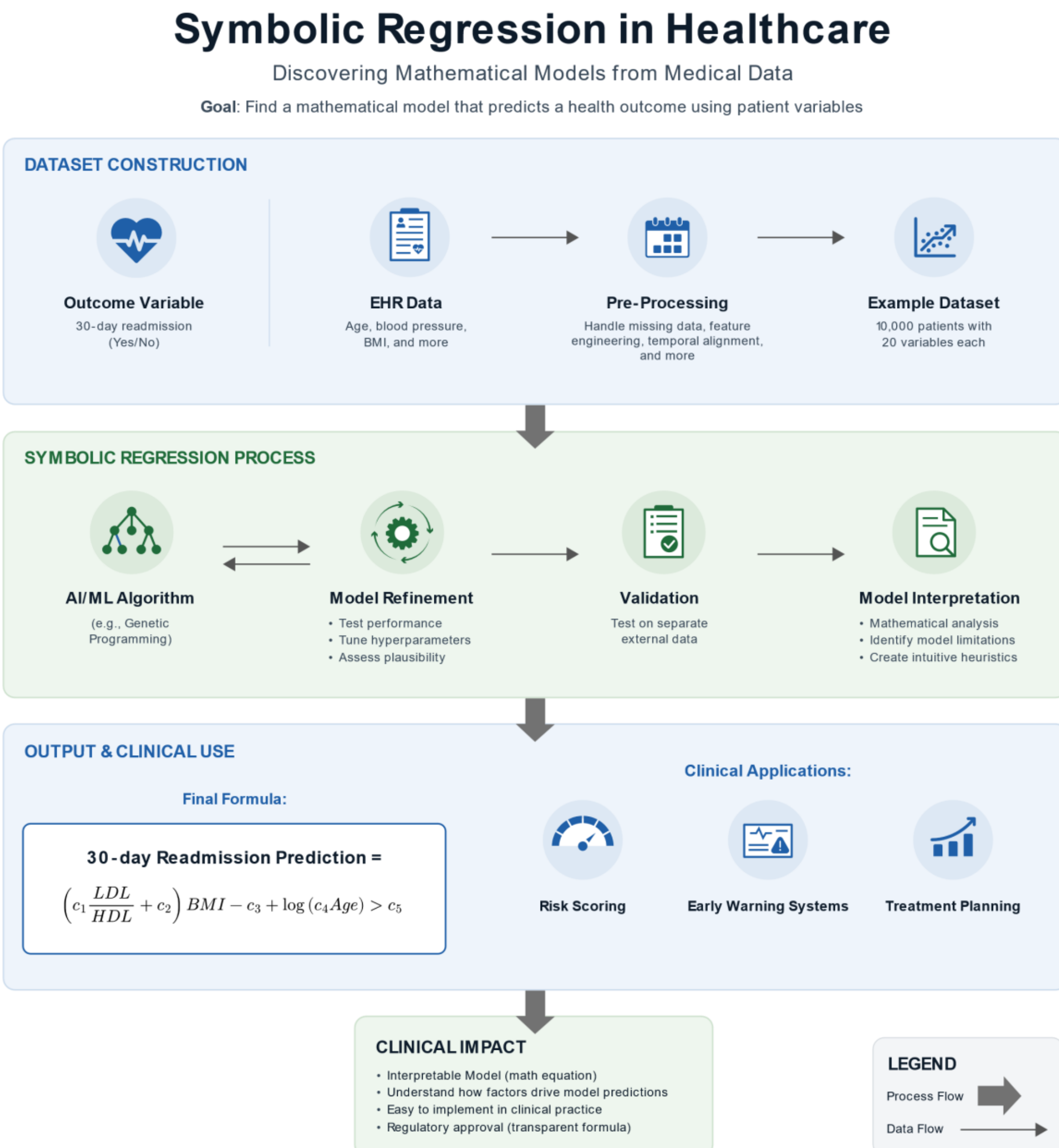

Figure 2: Example scenario to illustrate SR modeling with healthcare data.

5. In addition, I think the manuscript could engage more explicitly with the fact that, in many real-world prediction problems, a large number of different models often achieve very similar predictive performance. This point has been discussed prominently by Cynthia Rudin and others, for example in the context of the Rashomon set. From this perspective, symbolic regression should perhaps be positioned more clearly as one promising option

within a broader set of similarly performing models, rather than implicitly as a method that resolves most existing limitations.

We appreciate this excellent suggestion. We clarify that SR should not be considered a quest for a single "optimal" expression. Instead, we acknowledge that multiple models may achieve near-optimal performance. This perspective supports a more deliberate focus on interpretability. Also, working with high-dimensional data, we can be bold in applying domain-specific constraints. We added the following paragraphs to lines 160-173 to make these point clear, and cite Liu and Rudin:

> "It has been noted in the data science literature that for any given data set, many different models will provide near-optimal performance (the Rashomon effect[24]). Liu and Rudin define a data set's Rashomon set as the set of all mathematical functions (i.e., trained models) achieving a goodness of fit within acceptable bounds[24,25]. The task of constructing interpretable models can then be reframed as discovering the most desirable and interpretable functions within the Rashomon set. They argue that this reframing allows us to be bold in our pursuit of interpretability and that we should not shy away from imposing model constraints, such as sparsity. Furthermore, they envision this search as an interactive process, with data scientists iteratively improving on their models through experimentation.
>
> SR aligns very well with this approach. User-defined constraints and primitive functions allow for exploration of a space of human-readable models belonging to different function classes. One may consider coupling SR with a co-design mechanism for human involvement during the iterative, learning process[16]. Domain experts can review each equation and gauge model performance."

6. More generally, at several points the commentary reads as if symbolic regression might be a broadly superior solution to current challenges in interpretable machine learning and statistical modelling. I would encourage the authors to soften this impression. As emphasized repeatedly in the methodological literature, there is no "one method fits all" solution. This has been articulated clearly, for example, by Strobl and Leisch ("Against the 'one method fits all data sets' philosophy for comparison studies in methodological research", DOI: 10.1002/bimj.202200104) as well as by Anne-Laure Boulesteix and colleagues. Making this perspective more explicit would help to present a more balanced and realistic view.

We appreciate this concern. We have modified the language so that it does not give the impression that SR is superior to other interpretable ML models. We also include a statement that there is no "one method fits all" for ML in healthcare in the text of the previous response and cite Strobl and Leisch as well as Anne-Laure Boulesteix (lines 288-297). We hope that our

presentation of SR may help offer researchers more information on what to expect from this approach, and why SR may differ from other ML methods when applied to healthcare problems. This may help researchers (and in the future, policy-makers and healthcare organization leadership) make informed decisions when selecting methods for model building.

> "Lastly, no single analytic method fits all data. In practice, non-exhaustive algorithms are biased in which models they discover in their search space. SR models can miss desirable solutions during the training process. Additionally, SR algorithms, particularly genetic algorithms, are considered high variance, where small changes in training data result in very different solutions[41]. Finding the right constraints or hyperparameters to steer the final model away from undesirable solutions remains a challenge.
>
> SR represents only one of many interpretable methods available (e.g., rule mining and sparse linear regression). One should consider all options when selecting an approach to model building. SR remains scarcely tested in healthcare, and as new applications emerge, we must be wary of "over-optimism"[42]."

In addressing language choice, we have altered our introduction. The altered text on lines 32-37 presents SR as a method which may occupy a niche in medical ML.

> "In optimal circumstances, SR can produce models with both interpretability comparable to classical regression models and performance competitive with state-of-the-art tree-based and deep learning models[1,3]. In healthcare, ML implementation incorporating SR may offer opportunities to enhance performance while maintaining transparency and patient safety[4]. However, its value over other ML approaches remains unproven."

Furthermore, we have altered our section on data efficiency (lines 174-181) to soften the claim of SR outperforming other models on small data sets. We instead note the existence of evidence that SR performs well on small data sets and present it as a noteworthy method when large data sets are not available.

> ***"Data Efficiency***
>
> Symbolic regression has demonstrated good performance on small datasets. Wilstrup and Kasak found that SR outperformed classical regression and tree-based models across 240 datasets of 250 points or less[26]. This may make SR an appealing option when data is not readily available or is difficult to collect. For example, SR has been successfully applied to modeling electrical flow based on power, voltage, and current with only a few hundred samples[27]. However, such applications typically involve only a few underlying principles or laws, so applicability to other complex systems and relationships remains to be seen."

Finally, we alter our conclusion to present SR as a suitable option for model building in healthcare rather than an advancement over existing techniques. Additionally, we note that models should be externally validated before being considered (lines 408-418).

> "**Conclusion**
>
> Symbolic regression addresses the concern of machine learning model interpretability. Although its performance can vary, it offers the potential for better performance and generalizability, particularly when data size is small. SR may be considered in healthcare data modeling, recognizing its limitations and the need for domain knowledge.
>
> Unlike "black box" ML, SR generates transparent, human-readable equations. This is invaluable in clinical settings, where clinicians can use equations to interpret risk and make informed decisions. SR may offer insights on mechanistic relationships, such as between lifestyle and physiology. Health systems may consider the adoption of externally verified SR models utilizing EHR data given the ubiquitous concerns of trust and opaqueness of current, difficult-to-interpret algorithms."

Referee #3 (Remarks to the Author):

1. … its applicability in healthcare is made less clear. The main argument the authors give that symbolic regression is applicable to medicine is twofold: (1) Medical datasets are used as benchmarks for symbolic regression algorithms and (2) examples from recent literature of successful application of symbolic regression. It begs the question: If symbolic regression is already being successfully applied in healthcare, would a review of those methods be more appropriate? What is the novelty of this manuscript?

We thank the referee for this important point. Very little has been published on SR applied in healthcare. Only a few studies have utilized EHR data, with La Cava et al. noting that they are the first to do so in their 2023 publication[45]. This is pioneering work, with little accomplished towards external validation or deployment. At the same time, we also discovered that the knowledge gained from SR application in other domains (e.g., physics and engineering) have substantially contributed to what we know about SR from those experiences. To our knowledge, this is the first introductory commentary on SR addressed specifically to healthcare informaticists and researchers, drawing on validated knowledge from physics and engineering. We provide an approachable introduction to SR and describe its advantages and disadvantages, potential applications to healthcare, and SR-specific needs for future deployment and implementation in practice.

2. The examples of symbolic regression (e.g. interpolation and extrapolation, figure 1) are

didactic, but mathematically trivial. It would really help the stated purpose of the manuscript if examples taken were from medicine.

We hoped this introduction may be instructive for the target audience (informaticists and researchers) to conceptually appreciate interpolation and extrapolation with SR, without mathematical complexity.

- We have further developed the text that explains Figure 1 to provide easier interpretation (see Referee 1, #5).
- We now provide an example from medicine, and have developed a new schematic, Figure 2 (see Referee 2, #4).

3. The authors frame symbolic regression as a method for finding the "true or approximate functional form of the ground truth". They do not clearly explain what this means in a medical context. It is also unclear from the text if symbolic regression is being presented as a modeling technique (where the task is to find a mathematical representation which matches observations well enough for some purpose) or discovering a previously unknown true underlying functionality.

A "ground truth" may be better appreciated in other domains, such as physics, where there are clearly defined laws that are precisely formalized with mathematical equations. In contrast, this may be less applicable to predictive or diagnostic models in healthcare. In this case, data are more complex, noisy, incomplete, biased, and probabilistic. Thus, we believe that SR in healthcare would be more of a modeling technique, without attempting to discover some formula representing a "ground truth."

On lines 72-75, we reframe the objective of SR as aiming to find an idealized model consistent with underlying phenomena or an approximation of one.

> "The goal of SR is to identify (or approximate) an idealized model consistent with the underlying mechanisms of the system being modeled. It accomplishes this by constructing the model's functional form dynamically and optimizing a small number of constants, rather than relying on many weights and nested simple functions, as is the case with black-box networks."

In our description of the training process of transformer-based SR models, we replace "ground truth" with "true function" on lines 100-102:

> "These transformers are pre-trained on datasets generated from a wide variety of mathematical equations using token-level cross-entropy (with the true function as a loss function)."

On line 150-152, we have also replaced the interpretability benefit of gleaning insight into the underlying mechanisms:

> "It offers the ability to manually compute and understand output, predict behavior from distribution data, and glean insight into the underlying mechanisms of the process being modeled."

Also, the modifications made for Referee 2 #5 support how SR-derived models are one of many potentially suitable solutions, rather than a single, "true" model.

Lastly, we replaced the term "ground truth" with "function used to generate the data" in our expanded methodology for Figure 1 (see Referee 1, #5).

4. Finally, on framing: The manuscript is described as a commentary, but reads more like a tutorial on symbolic regression.

We have modified the text based on the referee comments, which we believe will make this appear more as a commentary. We introduced SR in the healthcare context and provided perspectives on its potential impacts and limitations, including policy and clinical implementation issues. We draw attention to implementation concerns with the addition of text related to fairness, bias, and clinical validation. We present unique implications and raise questions for future directions and explorations.

We do appreciate that some text resembles a tutorial. As our target audience (e.g., informaticists, researchers) is unlikely to be familiar with SR, we hoped to provide a meaningful introduction to SR. We hope this might encourage others to consider SR in their modeling research. As very little has been published in healthcare using SR, this offers a novel look at a new direction. If there are further suggestions, we remain open to additional modifications.

Sincerely,

Michael Ferguson, PhD

Post-Doctoral Fellow

Cardiovascular Digital Health Research (CODER) Training Program

UMass Chan Medical School

References:

1. Dong, J. & Zhong, J. Recent Advances in Symbolic Regression. *ACM Comput Surv* **57**, 290:1-290:37 (2025). **Dong & Zhong provide a thorough review of foundational and contemporary symbolic regression techniques, discussing their history, how they function, their strengths and weaknesses, relevant software tools, and examples of where they see use.**
2. Angelis, D., Sofos, F. & Karakasidis, T. E. Artificial Intelligence in Physical Sciences: Symbolic Regression Trends and Perspectives. *Arch. Comput. Methods Eng.* **30**, 3845–3865 (2023).
3. Aldeia, G. S. I. & de França, F. O. Interpretability in symbolic regression: a benchmark of explanatory methods using the Feynman data set. *Genet. Program. Evolvable Mach.* **23**, 309–349 (2022).
4. Ennab, M. & Mcheick, H. Enhancing interpretability and accuracy of AI models in healthcare: a comprehensive review on challenges and future directions. *Front. Robot. AI* **11**, 1444763 (2024).
5. Lemos, P., Jeffrey, N., Cranmer, M., Ho, S. & Battaglia, P. Rediscovering orbital mechanics with machine learning. *Mach. Learn. Sci. Technol.* **4**, 045002 (2023).
6. Morales-Alvarado, M., Conde, D., Bendavid, J., Sanz, V. & Ubiali, M. Symbolic regression for precision LHC physics. Preprint at https://doi.org/10.48550/arXiv.2412.07839 (2024).
7. Shojaee, P. *et al.* LLM-SRBench: A New Benchmark for Scientific Equation Discovery with Large Language Models. Preprint at https://doi.org/10.48550/arXiv.2504.10415 (2025).

8. La Cava, W. *et al.* Contemporary Symbolic Regression Methods and their Relative Performance. *Proc. Neural Inf. Process. Syst. Track Datasets Benchmarks* **1**, (2021).
9. Sotiropoulos, D. N., Koronakos, G. & Solanakis, S. V. Evolving Transparent Credit Risk Models: A Symbolic Regression Approach Using Genetic Programming. *Electronics* **13**, 4324 (2024).
10. Li, Q. *et al.* Advancing symbolic regression for earth science with a focus on evapotranspiration modeling. *Npj Clim. Atmospheric Sci.* **7**, 321 (2024).
11. Abdellaoui, I. A. & Mehrkanoon, S. Symbolic regression for scientific discovery: an application to wind speed forecasting. in *2021 IEEE Symposium Series on Computational Intelligence (SSCI)* 01–08 (2021). doi:10.1109/SSCI50451.2021.9659860.
12. Franca, F. O. de *et al.* Interpretable Symbolic Regression for Data Science: Analysis of the 2022 Competition. Preprint at https://doi.org/10.48550/arXiv.2304.01117 (2023).
13. Cranmer, M. Interpretable Machine Learning for Science with PySR and SymbolicRegression.jl. Preprint at https://doi.org/10.48550/arXiv.2305.01582 (2023).
14. Wang, L., Wu, Z., Sun, K., Li, Z. & Cheng, R. EvoGP: A GPU-accelerated Framework for Tree-Based Genetic Programming. Preprint at https://doi.org/10.48550/arXiv.2501.17168 (2025).
15. Petersen, B. K. *et al.* Deep symbolic regression: Recovering mathematical expressions from data via risk-seeking policy gradients. Preprint at https://doi.org/10.48550/arXiv.1912.04871 (2021).

16. Tian, Y. *et al.* Interactive symbolic regression with co-design mechanism through offline reinforcement learning. *Nat. Commun.* **16**, 3930 (2025).

17. Shojaee, P., Meidani, K., Barati Farimani, A. & Reddy, C. Transformer-based Planning for Symbolic Regression. *Adv. Neural Inf. Process. Syst.* **36**, 45907–45919 (2023).

18. Alaa, A. M. & van der Schaar, M. Demystifying Black-box Models with Symbolic Metamodels. in *Advances in Neural Information Processing Systems* (eds Wallach, H. et al.) vol. 32 (Curran Associates, Inc., 2019).

19. Jin, Y., Fu, W., Kang, J., Guo, J. & Guo, J. Bayesian Symbolic Regression. Preprint at https://doi.org/10.48550/arXiv.1910.08892 (2020).

20. Landajuela, M. *et al.* A Unified Framework for Deep Symbolic Regression. *Adv. Neural Inf. Process. Syst.* **35**, 33985–33998 (2022).

21. Albano, G., Giorno, V., Román-Román, P. & Torres-Ruiz, F. Study of a general growth model. *arXiv.org* https://arxiv.org/abs/2402.00882v1 (2024) doi:10.1016/j.cnsns.2021.106100.

22. Goethals, S., Martens, D. & Evgeniou, T. Manipulation Risks in Explainable AI: The Implications of the Disagreement Problem. in *Machine Learning and Principles and Practice of Knowledge Discovery in Databases* (eds Meo, R. & Silvestri, F.) 185–200 (Springer Nature Switzerland, Cham, 2025). doi:10.1007/978-3-031-74633-8_12.

23. Krishna, S. *et al.* The Disagreement Problem in Explainable Machine Learning: A Practitioner's Perspective. Preprint at https://doi.org/10.48550/arXiv.2202.01602 (2024).

24. Breiman, L. Statistical Modeling: The Two Cultures (with comments and a rejoinder by the author). *Stat. Sci.* **16**, 199–231 (2001).

25. Liu, J. & Rudin, C. User-Guided Interpretable Models: Rashomon Effect, Interaction, and Computation. *Harv. Data Sci. Rev.* **7**, (2025).

26. Wilstrup, C. & Kasak, J. Symbolic regression outperforms other models for small data sets. Preprint at https://doi.org/10.48550/arXiv.2103.15147 (2021).

27. Eichhorn, S., Mohapatra, A. & Goebel, C. PISR: Physics-Informed Symbolic Regression for Predicting Power System Voltage. in *Proceedings of the 16th ACM International Conference on Future and Sustainable Energy Systems* 92–107 (Association for Computing Machinery, New York, NY, USA, 2025). doi:10.1145/3679240.3734622.

28. Cho, S., Kim, D. & Hazlett, C. Inference at the data's edge: Gaussian processes for modeling and inference under model-dependency, poor overlap, and extrapolation. Preprint at https://doi.org/10.48550/arXiv.2407.10442 (2024).

29. Ziyin, L., Hartwig, T. & Ueda, M. Neural Networks Fail to Learn Periodic Functions and How to Fix It. in *Advances in Neural Information Processing Systems* vol. 33 1583–1594 (Curran Associates, Inc., 2020).

30. Li, W. *et al.* A Neural-Guided Dynamic Symbolic Network for Exploring Mathematical Expressions from Data. Preprint at https://doi.org/10.48550/arXiv.2309.13705 (2024).

31. Sahoo, S., Lampert, C. & Martius, G. Learning Equations for Extrapolation and Control. in *Proceedings of the 35th International Conference on Machine Learning* 4442–4450 (PMLR, 2018).

32. Ferrari, D., Guidetti, V., Wang, Y. & Curcin, V. Multi-objective Symbolic Regression to Generate Data-driven, Non-fixed Structure and Intelligible Mortality Predictors using EHR: Binary Classification Methodology and Comparison with State-of-the-art. *AMIA. Annu. Symp. Proc.* **2022**, 442–451 (2023).

33. Russeil, E. *et al.* Multi-View Symbolic Regression. Preprint at https://doi.org/10.48550/arXiv.2402.04298 (2024).

34. Hasanzadeh, F. *et al.* Bias recognition and mitigation strategies in artificial intelligence healthcare applications. *Npj Digit. Med.* **8**, 154 (2025).

35. Imai Aldeia, G. S. *et al.* Call for Action: towards the next generation of symbolic regression benchmark. in *Proceedings of the Genetic and Evolutionary Computation Conference Companion* 2529–2538 (Association for Computing Machinery, New York, NY, USA, 2025). **Aldeia et al. assess a wide range of state-of-the-art SR algorithms using modified benchmark problems, providing an objective overview of their current performances.**

36. Radwan, Y. A., Kronberger, G. & Winkler, S. A Comparison of Recent Algorithms for Symbolic Regression to Genetic Programming. in *Computer Aided Systems Theory – EUROCAST 2024* (eds Quesada-Arencibia, A., Affenzeller, M. & Moreno-Díaz, R.) 157–171 (Springer Nature Switzerland, Cham, 2025). doi:10.1007/978-3-031-82949-9_15.

37. Sun, C., Shen, S., Tao, W., Xue, D. & Zhou, Z. Noise-Resilient Symbolic Regression with Dynamic Gating Reinforcement Learning. *Proc. AAAI Conf. Artif. Intell.* **39**, 20690–20698 (2025).

38. Wang, X., Zhao, H., Zhao, Q. & Jiang, B. Deep Reinforcement Learning-Based Symbolic Regression for PDE Discovery Using Spatio-Temporal Rewards. in *2024 IEEE 20th International Conference on Automation Science and Engineering (CASE)* 3256–3261 (2024). doi:10.1109/CASE59546.2024.10711602.
39. Cao, X. & Yousefzadeh, R. Extrapolation and AI transparency: Why machine learning models should reveal when they make decisions beyond their training. *Big Data Soc.* **10**, 20539517231169731 (2023).
40. Epperson, J. F. On the Runge Example. *Am. Math. Mon.* **94**, 329–341 (1987).
41. Kammerer, L., Kronberger, G. & Winkler, S. Bias and Variance Analysis of Contemporary Symbolic Regression Methods. *Appl. Sci.* **14**, (2024).
42. Boulesteix, A.-L. Ten Simple Rules for Reducing Overoptimistic Reporting in Methodological Computational Research. *PLOS Comput. Biol.* **11**, e1004191 (2015).
43. DeCamp, M. & Tilburt, J. C. Why we cannot trust artificial intelligence in medicine. *Lancet Digit. Health* **1**, e390 (2019).
44. Arbelaez Ossa, L. *et al.* Re-focusing explainability in medicine. *Digit. Health* **8**, 20552076221074488 (2022).
45. La Cava, W. G. *et al.* A flexible symbolic regression method for constructing interpretable clinical prediction models. *Npj Digit. Med.* **6**, 1–14 (2023). **La Cava et al. conduct the first study utilizing SR for EHR prediction modeling and demonstrate the viability of their approach by performing a comprehensive analysis and validation of their methodology and models.**

46. Arina, P. *et al.* Mortality prediction after major surgery in a mixed population through machine learning: a multi-objective symbolic regression approach. *Anaesthesia* **80**, 551–560 (2025).

47. Ferrari, D. *et al.* Using interpretable machine learning to predict bloodstream infection and antimicrobial resistance in patients admitted to ICU: Early alert predictors based on EHR data to guide antimicrobial stewardship. *PLOS Digit. Health* **3**, e0000641 (2024).

48. Afzal, W. Search-based approaches to software fault prediction and software testing. (Mälardalen University, 2009).

49. Sorour, S. S., Saleh, C. A. & Shazly, M. Integrating machine learning and symbolic regression for predicting damage initiation in hybrid FRP bolted connections. *Sci. Rep.* **15**, 18564 (2025).

50. de Rooij, M., van Riel, N. A. W. & O'Donovan, S. D. Conditional universal differential equations capture population dynamics and interindividual variation in c-peptide production. *Npj Syst. Biol. Appl.* **11**, 84 (2025).

51. Hossain, M. A., Elmoselhi, H., Elshorbagy, A. A. & Shoker, A. The Sask formula to estimate glomerular filtration rate in renal transplant patients. *Nephron Clin. Pract.* **117**, c135-150 (2011).

52. Fong, K. S. & Motani, M. Symbolic Regression for Discovery of Medical Equations: A Case Study on Glomerular Filtration Rate Estimation Equations. in *2024 IEEE Conference on Artificial Intelligence (CAI)* 1373–1379 (2024). doi:10.1109/CAI59869.2024.00245.

53. Hidalgo, J. I. *et al.* Modeling glycemia in humans by means of Grammatical Evolution. *Appl. Soft Comput.* **20**, 40–53 (2014).

54. Chiavegatto Filho, A., Batista, A. F. D. M. & dos Santos, H. G. Data Leakage in Health Outcomes Prediction With Machine Learning. Comment on “Prediction of Incident Hypertension Within the Next Year: Prospective Study Using Statewide Electronic Health Records and Machine Learning”. *J. Med. Internet Res.* **23**, e10969 (2021).

55. Han, L. Addressing Distribution Shift for Robust and Trustworthy Prediction and Causal Inference in Clinical AI Settings. *JAMA Netw. Open* **8**, e2513705 (2025).

56. Subasri, V. *et al.* Detecting and Remediating Harmful Data Shifts for the Responsible Deployment of Clinical AI Models. *JAMA Netw. Open* **8**, e2513685 (2025).

57. Warraich, H. J., Tazbaz, T. & Califf, R. M. FDA Perspective on the Regulation of Artificial Intelligence in Health Care and Biomedicine. *JAMA* **333**, 241–247 (2025).

58. Shah, N. H., Pfeffer, M. A. & Ghassemi, M. The Need for Continuous Evaluation of Artificial Intelligence Prediction Algorithms. *JAMA Netw. Open* **7**, e2433009 (2024).

59. Kotanchek, M. Answer to ‘Symbolic Regression’. *Mathematica Stack Exchange* https://mathematica.stackexchange.com/a/251478 (2021).

August 27, 2026

RE: Manuscript #: COMMSMED-25-3026A

Dear Referees,

We appreciate your continued thoroughness in evaluating our manuscript "**Introduction to Using Symbolic Regression for Interpretable Machine Learning in Healthcare**". We have taken these comments very seriously and hope they are adequately addressed.

For clarity, references highlighted in green represent changes made during the first round of revisions. Any modifications made during this second (current) round of revisions are highlighted in yellow. This decision was made after consulting with the editorial team of *Communications Medicine*.

Referee #1:

1. Thanks for addressing the comments but the credibility concerns remain. For instance, "Bahri et al" now became "Wilstrup and Kasak" and the change is not highlighted in the bibliography.

We sincerely apologize for this oversight. It appears that the highlighting was removed by our citation manager (Zotero) during the final generation of the bibliography. The revised source "*Symbolic Regression Outperforms Other Models on Small Data Sets*" by Wilstrup and Kasak has now been highlighted to indicate the change from the originally submitted manuscript (lines 516-517). We have also highlighted all other references that were added or modified after the original submission of the manuscript. Sections of the text highlighted in green represent changes that should have been highlighted during the *first* round of revisions (yellow highlighting represents modifications made with the *second*, current revision). Also, we note that reference 48 was revised to adhere to Nature style guidelines (the source itself has not changed). No new references were included, and no other references had their citation altered during this round of revisions.

Because of the credibility concerns raised, two of the authors have independently reviewed each reference in the bibliography. For each reference, we reviewed the source article and its contribution to the manuscript text where referenced. For thoroughness and due diligence, we now provide 1-2 summary sentences for each reference describing its contribution in a separate document (beyond the suggested 5-10% of references per journal instructions).

2. Lines 568 - 571 includes run-off text: "La Cava et al. conduct the first study utilizing SR for EHR prediction modeling and demonstrate the viability of their approach by performing a comprehensive analysis and validation of their methodology and models."

Similarly, lines 540-542 contain "Aldeia et al. assess a wide range of state-of-the-art SR algorithms using modified benchmark problems, providing an objective overview of their current performances."

We are uncertain as to the concern for "run-off text." The text "La Cava et al. conduct the first study utilizing SR…" and "Aldeia et al. assess a wide range of…" were intentionally added to the bibliography. The "Revision Instructions – Reviews & Perspectives" document we received from the editorial team included the following guidance for authors: "We recommend that you select the most significant 5–10% of references in your list for highlighting and add a single sentence in bold after each of these references to describe the main result and its significance."

For clarity, we have edited these sentences further:

- On lines 541-545 the sentence concerning the study by Aldeia et al. has been changed to "Aldeia et al. use modified benchmark problems to provide an objective overview of a wide range of state-of-the-art SR algorithms."
- On lines 569-572 the sentence concerning the study by La Cava et al. has been changed to "La Cava et al. conduct the first study utilizing SR for EHR prediction modeling and perform a thorough scientific analysis to demonstrate the viability of their approach."

<u>Referee #2:</u>

1. …The author correctly acknowledge in their answer that symbolic regression is most suitable for exploratory or prediction analyses but not for confirmatory data analyses. However, I did not find that statement in the manuscript. I think it would be good for the reader if this is also acknowledged there - please forgive me if I simply overlooked this.

We now include text in the manuscript that SR is most suitable for exploratory or prediction analyses, but not for confirmatory data analyses. We have added the following text to the section on limitations on lines 290-295:

> "Symbolic regression is most suitable for exploratory or prediction analyses, rather than confirmatory data analyses. SR is inherently data-driven and is particularly applicable in settings with uncertain or complex relationships where prespecified model structures are not available. Extensions to support confirmatory analyses, including uncertainty quantification and formal inference, may represent areas for future work."

Sincerely,

Michael Ferguson, PhD
Post-Doctoral Fellow

Cardiovascular Digital Health Research (CODER) Training Program
UMass Chan Medical School
